# Bioelectrical impedance-derived phase angle as a contextual indicator for steatotic liver disease and hepatic fat estimation

**DOI:** 10.3389/fnut.2026.1787419

**Published:** 2026-04-10

**Authors:** Güleren Sabuncular, Zehra Margot Çelik, Coşkun Özer Demirtaş, Şule Aktaç, Fatih Eren

**Affiliations:** 1 Faculty of Health Sciences, Department of Nutrition and Dietetics, Marmara University, Istanbul, Türkiye; 2 Department of Gastroenterology, School of Medicine, Marmara University, Istanbul, Türkiye; 3 School of Medicine, Recep Tayyip Erdogan University, Rize, Türkiye; 4 Department of Medical Biology, School of Medicine, Marmara University, Istanbul, Türkiye; 5 Liver Research Unit, Institute of Gastroenterology, Marmara University, Istanbul, Türkiye

**Keywords:** bioelectrical impedance analysis, controlled attenuation parameter, nutritional assessment, phase angle, predictive equations, steatotic liver disease

## Abstract

Steatotic liver disease (SLD) is highly prevalent among individuals with metabolic risk factors, highlighting the need for accessible, non-invasive tools that can support nutritional and clinical risk contextualization. Phase angle, derived from bioelectrical impedance analysis, reflects cellular integrity and body composition and is widely used in nutritional assessment. In this cross-sectional study, 495 adults underwent multi-frequency bioelectrical impedance analysis and hepatic fat evaluation using the controlled attenuation parameter obtained by FibroScan®. Participants were classified as having SLD based on a controlled attenuation parameter threshold of ≥275 dB/m. Individuals with SLD exhibited significantly higher whole-body and segmental phase angle values compared with those without SLD, and these differences were consistent across age and obesity subgroups (*p* < 0.05). Whole-body phase angle demonstrated moderate discriminative ability for SLD status (area under the curve = 0.646), supporting its role as a contextual indicator rather than a diagnostic measure. In multivariable logistic regression analyses, phase angle remained independently associated with SLD alongside waist circumference and age, whereas body mass index and body fat percentage were not statistically significant. Additionally, a regression-based model incorporating phase angle and simple anthropometric variables showed good performance in estimating hepatic fat content (area under the curve = 0.807). Overall, these findings indicate that phase angle reflects bioelectrical properties influenced by body composition associated with clinically relevant hepatic steatosis.

## 1 Introduction

Steatotic liver disease (SLD), encompassing both metabolic dysfunction-associated steatotic liver disease (MASLD) and alcohol-related liver disease (ALD), represents a major global health burden, collectively affecting, based on 2019 Global Burden of Disease estimates, approximately 1.25 billion individuals worldwide (1, 2). Hepatic steatosis, whether resulting from excessive alcohol consumption or metabolic dysfunction, is a critical initial step that may advance to steatohepatitis and its complications such as cirrhosis or hepatocellular carcinoma (HCC) (2, 3). The predominantly asymptomatic and non-specific presentation of early-stage hepatic steatosis often leads to delayed diagnosis and missed opportunities for timely intervention (1, 2). Therefore, timely identification and appropriate risk contextualization are essential for preventing complications and improving clinical outcomes (3).

Although liver biopsy is currently considered the gold standard for detecting steatosis and fibrosis, its use is limited due to its invasive nature, high cost, and risk of complications (4). In this context, there is an increasing demand for less invasive, cost-effective, and more accessible assessment tools. In recent years, the Controlled Attenuation Parameter (CAP), measured via vibration-controlled transient elastography (FibroScan®), has emerged as a method for quantifying liver fat based on the attenuation properties of ultrasonic signals. CAP measurements have gained increasing clinical utility due to their ability to assess a larger volume of liver tissue compared to biopsy and to reduce sampling error (5).

Bioelectrical impedance analysis (BIA) is a widely used, non-invasive method for evaluating body composition. It operates by applying a low-amplitude electrical current to the body and measuring the resulting resistance, which reflects the conductivity characteristics of tissues and allows estimation of fat, muscle, and water content. Since the current primarily flows through extracellular fluids, reduced body water content increases resistance, enabling assessment of hydration status (6). When the electrical current encounters the cell membranes of tissues (fat, muscle) within different body compartments, a portion of the current is stored by the membranes, causing a phase shift in the current’s flow (7). This shift is measured as reactance (Xc). Reactance is highly variable and depends on the integrity and composition of the cell membrane (7, 8).

The Phase Angle (PhA) is derived from the relationship between the resistive component (primarily from water/electrolyte conductivity) and the reactive component (stemming from the membrane’s capacitive effect). Specifically, the ratio of reactance to resistance yields the Phase Angle: {PhA} = arctan (Xc/R) (9, 10). A higher Phase Angle occurs when cell membranes exhibit better electrical storage capacity (higher reactance). This condition is generally associated with better cellular integrity and body cell mass (8, 9).

High fat and high carbohydrate diet and alcohol consumption are significant etiological factors in the pathogenesis of hepatic steatosis. Obesity and Type 2 Diabetes are also considered important comorbidities (11, 12). It is well-established that both advanced age and obesity are associated with lower phase angle values. Age-related declines in cellular membrane function and loss of lean body mass contribute to this reduction (13), while increased adiposity and altered fluid distribution in individuals with obesity can also affect PhA measurements (14). Although these physiological changes influence PhA, it is unclear whether the association between PhA and liver fat persists independently of age and body mass index (BMI). Recent research suggests that PhA may still serve as a useful biomarker of hepatic steatosis across different subgroups, regardless of body size or age (15). Deviations in PhA may be present even before abnormalities in other markers of malnutrition become evident. Considering this and the strong association between nutritional status and survival, European guidelines on the management of patients with liver disease recommend regular assessment of nutritional status using BIA (16). In this context, evaluating PhA through BIA as an indicator of nutritional status raises the question of its potential utility as a supportive, non-invasive marker within clinical and nutritional risk assessment for SLD.

In the literature, PhA has been investigated as a prognostic marker in various fields including cancer (17), respiratory diseases (18), and cardiovascular conditions (19). Although PhA has been shown to be an independent prognostic factor in cirrhosis (20), its role in earlier stages of liver disease, particularly SLD, remains underexplored.

Studies investigating the association between PhA and SLD are limited, and no standardized cut-off values have yet been established for its clinical application (21). Nevertheless, comprehensive research is needed to better understand the clinical relevance and contextual value of PhA. Rather than functioning as a standalone diagnostic test, its potential contribution within clinical and nutritional assessment frameworks warrants further investigation. Our study contributes to this growing body of evidence by evaluating the association of PhA with SLD in individuals with and without obesity, and in both younger and older adults. The present study aims to explore the potential utility of PhA, together with simple demographic and body composition parameters, as a supportive tool for identifying individuals at risk of SLD, particularly in clinical settings where access to advanced imaging techniques such as vibration-controlled transient elastography is limited. Specifically, the study had two complementary objectives: first, to evaluate whether PhA and body composition parameters obtained from bioelectrical impedance analysis could help identify individuals with SLD; and second, to explore whether liver fat levels reflected by CAP measurements could be approximated using these easily obtainable parameters.

## 2 Materials and methods

### 2.1 Participants

This retrospective study was conducted by screening patients who applied to the Gastroenterology Institute of Marmara University between 2022 and 2024. Prior to the initiation of the study, ethical approval was obtained from the Non-Interventional Clinical Research Ethics Committee of Recep Tayyip Erdoğan University Faculty of Medicine (Approval No: 2025/319).

The study included individuals aged 18 years and older who had undergone both FibroScan® and bioelectrical impedance analysis. Patients with a diagnosis of cancer, those who were pregnant or breastfeeding, and those diagnosed with cirrhosis were excluded from the study. Data were obtained from a total of 495 patient records.

This study was conducted in accordance with the principles of the Declaration of Helsinki. Informed consent was obtained from all subjects involved in the study.

### 2.2 Data collection

As part of the study, the following information was extracted from patient records: sociodemographic data (age, sex), information regarding the presence of chronic diseases (such as diabetes and hypertension), anthropometric measurements (height, body weight, body mass index, waist, hip and neck circumference), body composition analysis (body fat percentage, fat mass, muscle mass, fat-free mass, and total body water), and FibroScan results [CAP, dB/m] value, liver stiffness measurement (e, kPA) stage and interquartile range/median [IQR/MED] values.

### 2.3 Anthropometric measurements

Although data for this study were collected retrospectively, all anthropometric measurements had been conducted using standardized procedures at the time of patient evaluation. Body weight was measured using a Tanita 780MA bioelectrical impedance analysis (BIA) device. Prior to stepping onto the device, participants’ clothing weight was estimated and entered into the system for tare adjustment, and they were instructed to remove all metal items, shoes, and socks. Measurements were taken with clean and dry feet, ensuring proper contact of hands and heels with the electrodes.

Height was measured using a portable stadiometer. Participants were asked to remove accessories such as hairbands or clips, as well as their shoes. Before recording height, proper posture was confirmed: the back and shoulders were kept straight, the participant’s gaze was level and parallel to the ground, and the head, shoulders, buttocks, calves, and heels were aligned with the stadiometer. Feet were positioned side by side, slightly apart, and knees fully extended. Measurements were taken in the Frankfurt plane and recorded to the nearest 0.1 cm.

Body mass index (BMI) was calculated using the formula: weight (kg) / height^2^ (m^2^). The World Health Organization (22) classification was used to interpret BMI. The patients that had a BMI < 30 kg/m2 were classified as non-obese while patients that had a BMI ≥ 30 kg/m^2^ were classified as obese.

Waist circumference was measured by trained researchers while the participant was standing, using a non-stretchable measuring tape placed midway between the lower margin of the last palpable rib and the top of the iliac crest. Hip circumference was measured at the widest portion of the buttocks using a non-stretchable tape.

In accordance with the routine standardized procedures of the Gastroenterology Institute, all anthropometric and BIA measurements were performed once unless an unexpected measurement error was detected.

### 2.4 Body composition measurements

All body composition measurements had been performed using standardized protocols in clinical practice. A Tanita 780MA bioelectrical impedance analysis (BIA) device was used to assess body weight and body composition. This multi-frequency segmental BIA device is widely used in clinical and research settings for the assessment of body composition and impedance-derived parameters. At the time of measurement, participants were instructed to follow BIA preparation guidelines, including at least 4 h of fasting, avoidance of intense physical activity for 24–48 h prior, and postponement of measurement during menstruation, in accordance with established recommendations (10, 23). Measurements were performed with participants in a standing position, barefoot and wearing light clothing to minimize potential variability related to hydration status and recent physical activity.

From the BIA reports, the following parameters were recorded: body fat percentage, fat mass, muscle mass, fat-free mass (FFM), and total body water. The BIA device was routinely maintained and calibrated according to the manufacturer’s recommendations as part of standard clinical practice.

### 2.5 Phase angle calculation

Phase angle (PhA) values had been calculated at the time of assessment using standardized procedures. The BIA device measured segmental impedance for the right arm, left arm, right leg, left leg, and trunk across six frequencies. Reactance (Xc) and resistance (R) values at different frequencies were recorded in the analysis reports.

In the present study, raw impedance values obtained from the BIA device were used for phase angle calculation. Because the device provides impedance measurements at multiple frequencies, the reactance (Xc) and resistance (R) values measured at 50 kHz were selected to derive phase angle values, consistent with common practice in bioelectrical impedance analysis studies.

Phase angle represents the arctangent of the ratio between reactance and resistance, reflecting the relationship between capacitive and conductive properties of biological tissues. PhA was calculated for each body segment at 50 kHz using the formula (9, 10):

PhA=arctan(Xc/R)×180/π

### 2.6 Vibration-controlled transient elastography (FibroScan®)

Hepatic steatosis and liver stiffness data were obtained retrospectively from clinical assessments performed with a FibroScan® device using vibration-controlled transient elastography. All measurements had been performed by a hepatology specialist at the Gastroenterology Institute of Marmara University. The liver stiffness measurement (LSM) values ranged from 2.5 to 75 kPa, while the CAP, which reflects the degree of hepatic steatosis, ranged from 100 to 400 dB/m (24). In this study a CAP value ≥ 275 dB/m was used to define SLD, according to the EASL guidelines (25). CAP and fibrosis scores were obtained from the FibroScan reports recorded by the attending gastroenterologist. Due to the retrospective design of the study and incomplete documentation of alcohol consumption in the clinical records, reliable differentiation between SLD subtypes (MASLD, ALD, or MetALD) was not feasible. Therefore, the broader term SLD was used throughout the study.

### 2.7 Statistical analysis

All statistical analyses were conducted using IBM SPSS Statistics (version 29.0), and all graphical representations, were generated using R software (version 2025.05.01) with the ggplot2 package. Descriptive statistics for continuous variables were presented as means and standard deviations (SD), while categorical data were summarized as frequencies and percentages. A *p*-value < 0.05 was considered statistically significant.

Group differences in whole-body and segmental PhA values between participants with and without SLD were assessed using independent samples t-tests. To explore crude associations between CAP and selected continuous variables (PhA, BMI, waist circumference, body fat percentage, and age), Pearson correlation coefficients were calculated as an initial exploratory analysis.

To identify predictors of SLD (binary outcome: 0 = No, 1 = Yes), binary logistic regression analysis was employed. The model included PhA, BMI, waist circumference, body fat percentage, and age as independent variables. Results were reported as odds ratios (ORs) with 95% confidence intervals (CIs). For the logistic regression model predicting SLD, model diagnostics included assessment of multicollinearity using variance inflation factors (VIF), evaluation of the linearity of the logit for continuous predictors using the Box-Tidwell test, assessment of model fit using the Hosmer–Lemeshow test, discrimination using receiver operating characteristic (ROC) curve analysis, and examination of influential observations using Cook’s distance.

To develop a simplified regression model for predicting CAP values, we employed a linear regression approach focusing on a reduced set of predictors. For the linear regression models predicting CAP values, assumptions of linearity between predictors and outcome, homoscedasticity, normality of residuals, and absence of multicollinearity were assessed via visual inspection of residual plots and formal statistical testing. The regression formula was derived to predict CAP values as the dependent variable (y), with the in-dependent variables (x1,x2,…,xk) defined as follows:

CAP=β0+(β1×x1)+(β2×x2)+(β3×x3)….(βk×xk)

To evaluate the discriminatory ability of PhA in relation to SLD status, a receiver operating characteristic (ROC) curve was generated. The area under the curve (AUC) was calculated as a measure of classification performance, and the optimal cutoff point was determined using Youden’s Index, with corresponding sensitivity and specificity values reported.

## 3 Results

The demographic and clinical characteristics of the study participants are presented in Table 1. Of the total sample (*n* = 495), 54.5% were male, and the mean age was 47.0 ± 11.6 years. Based on FibroScan assessments, 53.9% of the participants were found to have hepatic steatosis. According to the BMI classification, 43.6% of the participants were categorized as obese.

**Table 1:** Demographic and clinical characteristic of the participants (*n* = 495).

Characteristic	*n* (%)/mean ± SD
Gender (Male), *n* (%)	270 (54.5)
Age (year)	47.0 ± 11.6
Height (cm)	167.0 ± 10.1
Weight (kg)	82.4 ± 17.8
BMI (kg/m2)	29.5 ± 6.0
Waist circumference (cm)	98.5 ± 15.3
Hip circumference (cm)	108.6 ± 11.6
Body fat mass (%)	29.8 ± 8.0
Muscle mass (kg)	54.4 ± 10.7
FFM (kg)	57.2 ± 11.2
Body water (kg)	41.6 ± 21.4
CAP (db/mL)	277.7 ± 59.4
PhA (o)	6.01 ± 0.79
LSM (kPa), median (min-max)	5.00 (2.30–75.00)
SLD, *n* (%)	
SLD	267 (53.9)
Non-SLD	228 (46.1)
Metabolic diseases, *n* (%)	
HTN	80 (16.2)
DM	71 (14.3)
HLD	47 (9.5)
IR	13 (2.6)
Obesity	216 (43.6)

BMI, body mass index; CAP, controlled attenuation parameter; DM, diabetes mellitus; FFM, fat-free mass; HLD, hyperlipidemia; HTN, hypertension; IR, insulin resistance; PhA, phase angle; SLD, steatotic liver disease. LSM, liver stiffness measurement.

The comparison of PhA (o) values of participants with and without SLD is shown in Table 2. For all the PhA (o) values it was found to be statistically significantly higher in participants with SLD (*p* < 0.001).

**Table 2:** Phase angle values according to SLD.

PhA (^o^)	SLD (*n* = 267)	Non-SLD (*n* = 228)	*p* value
Whole body PhA (^o^)	6.20 ± 0.78	5.80 ± 0.75	< 0.001
Right leg PhA (^o^)	5.88 ± 2.39	5.51 ± 0.84	0.014
Left leg PhA (^o^)	5.79 ± 0.93	5.53 ± 0.89	< 0.001
Right hand PhA (^o^)	6.64 ± 0.92	6.19 ± 0.81	< 0.001
Left hand PhA (^o^)	6.65 ± 1.12	6.15 ± 0.83	< 0.001

PhA, phase angle; independent sample *t*-test.

To further investigate whether the relationship between SLD and PhA is independent of age, participants were categorized based on age using a 40-year cut-off. In both age groups (<40 years and ≥40 years), individuals with SLD had significantly higher PhA values compared to those without (*p* < 0.001). Among participants under 40 years, the mean PhA was 6.50 ± 0.73 in those with SLD versus 5.88 ± 0.76 in those without SLD. Similarly, among those aged 40 and older, the mean PhA was 6.10 ± 0.77 in the SLD group versus 5.77 ± 0.74 in the non-SLD group. In addition, Pearson correlation analysis revealed a significant negative relationship between PhA and age in both groups. Among non-SLD participants, PhA was negatively correlated with age (*r* = −0.206, *p* = 0.002). This association was even stronger among those with SLD (*r* = −0.352, *p* < 0.001). A subgroup analysis by obesity status (BMI ≥ 30 kg/m^2^) showed that, regardless of obesity, participants with SLD had significantly higher PhA values than those without (*p* < 0.05). Among obese individuals, the mean PhA was 6.23 ± 0.75 in the SLD group versus 6.02 ± 0.81 in the non-SLD group. Among non-obese participants, the mean PhA was 6.14 ± 0.82 in the SLD group versus 5.73 ± 0.72 in the non-SLD group (not shown in table).

A comparison of demographic and anthropometric characteristics between the SLD and non-SLD groups is presented in Table 3. The prevalence of SLD was significantly higher among males (62.5%) compared with the non-SLD group (45.2%) (*p* < 0.001). Individuals with SLD were also older than those without SLD (48.8 ± 11.6 vs. 44.9 ± 11.3 years, *p* < 0.001). Anthropometric parameters showed markedly higher values in the SLD group. Participants with SLD had significantly greater BMI (31.9 ± 5.6 vs. 26.8 ± 5.3 kg/m^2^, *p* < 0.001), waist circumference (105.1 ± 13.3 vs. 90.7 ± 13.8 cm, *p* < 0.001), and body fat percentage (31.5 ± 7.6 vs. 27.7 ± 8.0%, *p* < 0.001) compared with the non-SLD group.

**Table 3:** Baseline characteristics of participants stratified by SLD status.

Variable	SLD (*n* = 267)	Non-SLD (*n* = 228)	*p* value
Gender, n (%)	Male: 167 (62.5) Female: 100 (37.5)	Male: 103 (45.2) Female: 125 (54.8)	< 0.001*
Age (year)	48.8 ± 11.6	44.9 ± 11.3	< 0.001**
BMI (kg/m^2^)	31.9 ± 5.6	26.8 ± 5.3	< 0.001**
Waist circumference (cm)	105.1 ± 13.2	90.7 ± 13.8	< 0.001**
Body fat (%)	31.5 ± 7.6	27.7 ± 8.0	< 0.001**
LSM (kPa)	5.70 (2.50–56.60)	4.40 (2.30–75.00)	< 0.001***
Fibrosis stage, *n* (%)			
No/Mild fibrosis (F0-F1)	187 (70.0)	207 (90.8)	< 0.001*
Significant fibrosis (F2)	48 (18.0)	9 (3.9)	
Advanced fibrosis (F3-F4)	32 (12.0)	12 (5.3)	

*Chi-square test, *n* (%); **Independent sample *T* test, mean±SD; *** Mann–Whitney U test, median (min-max); BMI, body mass index; LSM, liver stiffness measurement.

Fibrosis stage: No/mild fibrosis (F0-F1): <8 kPa; significant fibrosis (F2): 8–9.6 kPa; advanced fibrosis (F3-F4): >9.6.

When stratified by obesity status, 75.5% of the obese individuals were diagnosed with SLD, compared to 37.3% among non-obese individuals (*p* < 0.001) (not presented in table). Regarding liver stiffness assessment, participants with SLD had a significantly higher prevalence of significant-to-advanced fibrosis (≥ F2) compared with the non-SLD group (30.0% vs. 9.2%; *p* < 0.001).

**Figure 1:**
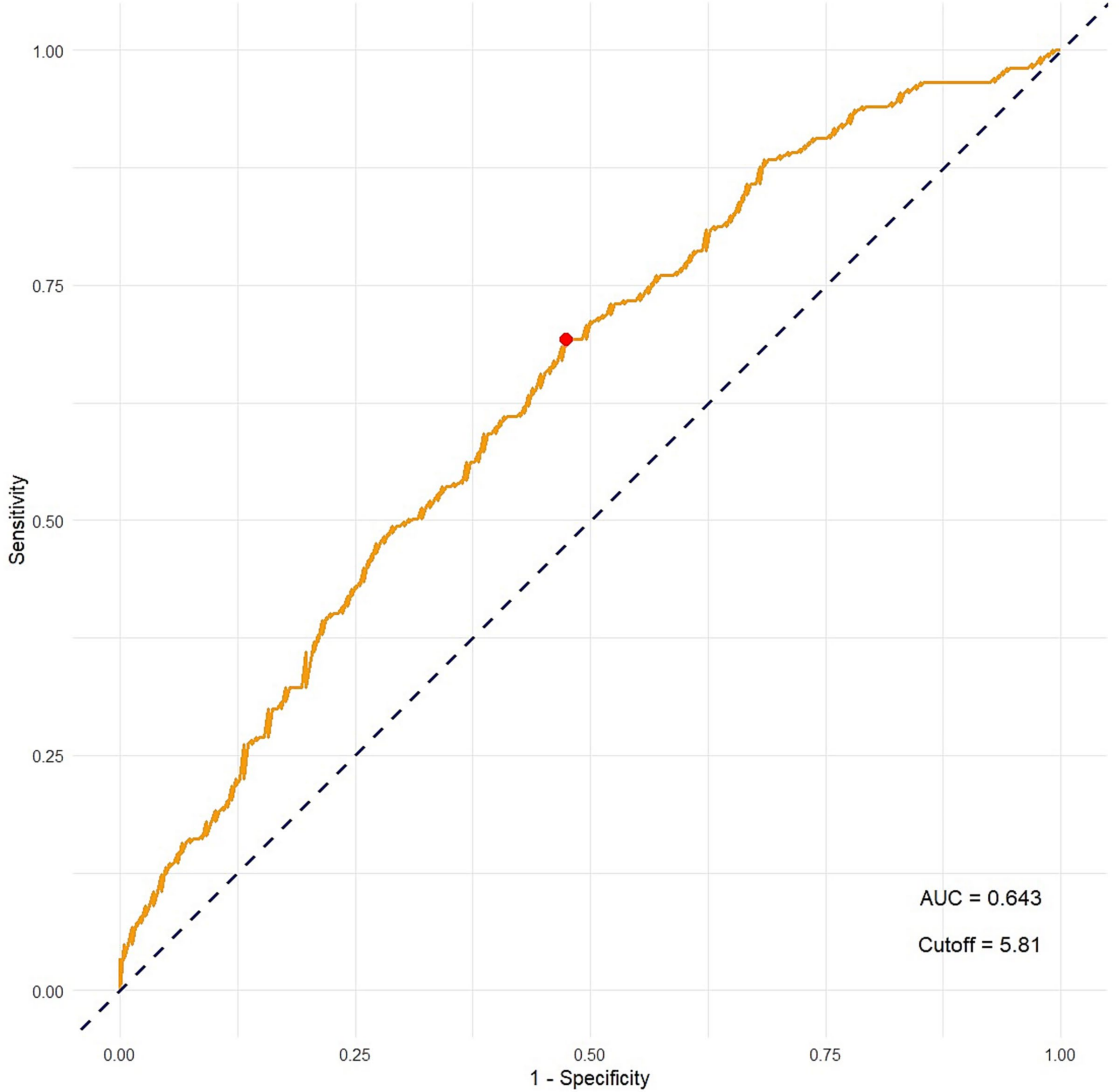
ROC curve evaluating the ability of phase angle (PhA) to discriminate SLD.

The area under the ROC curve (AUC) was 0.643, indicating a modest discriminative ability of PhA for identifying individuals with SLD. The optimal cut-off value, determined using Youden’s Index, was 5.810. At this threshold, the sensitivity was 0.693 and the specificity was 0.526. The positive predictive value (PPV) and negative predictive value (NPV) were 0.631 and 0.594, respectively. The positive likelihood ratio (LR+) was 1.463, and the negative likelihood ratio (LR−) was 0.583 (Figure 1).

Table 4 presents the results of the multivariable logistic regression model predicting SLD (CAP ≥ 275). The model included phase angle (PhA), BMI, waist circumference, age, and body fat percentage as predictors. The overall model was statistically significant (χ^2^ = 142.797, *p* < 0.001), with a Nagelkerke R^2^ of 0.335. The Hosmer–Lemeshow test indicated adequate model fit (*p* = 0.109), and the overall classification accuracy was 73.7%. Among the included variables, phase angle, waist circumference, and age were statistically significant predictors of SLD, whereas BMI and body fat percentage were not statistically significant in the adjusted model.

**Table 4:** Logistic regression model predicting SLD.

Variable	β (SE)	OR	95% CI	*p*-value
Whole Body PhA (^o^)	0.639 (0.193)	1.895	1.297–2.769	< 0.001
BMI (kg/m^2^)	0.028 (0.048)	1.028	0.936–1.130	0.558
Waist circumference (cm)	0.055 (0.014)	1.057	1.027–1.087	< 0.001
Age (year)	0.027 (0.010)	1.028	1.007–1.049	0.009
Body Fat (%)	0.020 (0.025)	1.020	0.972–1.071	0.416

BMI, body mass index; PhA, phase angle; OR, odds ratio; CI, confidence interval.

When the correlation between CAP value and various variables was examined, it was determined that there was a positive correlation with all the variables (*p* < 0.001) (Table 5). Among these variables, the strongest correlation was found between waist circumference and CAP value (*r* = 0.518; *p* < 0.001).

**Table 5:** Correlation between CAP value and various variables.

	Controlled attenuation parameter (CAP)	
Variables	*r*	*p*
Whole body PhA (^o^)	0.313	< 0.001
BMI (kg/m^2^)	0.447	< 0.001
Waist circumference (cm)	0.518	< 0.001
Body fat (%)	0.249	< 0.001
Age (year)	0.169	< 0.001

BMI, body mass index; PhA, phase angle; Pearson correlation.

Table 6 presents the results of linear regression analyses conducted to identify predictors of CAP values. Four models (M₁–M₄) were compared. Model 1 included only PhA, while Model 2 added waist circumference and body fat percentage. Model 3 incorporated age and gender, and Model 4 additionally included FFM and BMI. Model 3 demonstrated the best overall fit, with the highest adjusted R^2^ (0.310), lowest SEE (49.37), and lowest AIC (5273.08). Table 5 summarizes the unstandardized coefficients (*β*), standard errors (SE), and significance levels for each model, along with model fit statistics.

**Table 6:** Linear regression models predicting CAP values.

Predictor	Model 1 β (SE)	Model 2 β (SE)	Model 3 β (SE)	Model 4 β (SE)
Intercept	135.54 (19.59)***	9.75 (21.12)	9.64 (26.97)	17.47 (33.24)
Whole Body PhA (^o^)	23.64 (3.23)***	15.02 (3.25)***	15.93 (3.51)***	16.03 (3.70)***
WC (cm)	—	1.58 (0.19)***	1.02 (0.26)***	1.10 (0.33)***
Body Fat (%)	—	0.73 (0.34)*	1.73 (0.53)**	1.70 (0.71)*
Age (year)	—	—	0.63 (0.21)**	0.59 (0.22)**
Gender (female)	—	—	−20.06 (8.39)*	−23.11 (10.26)*
FFM (kg)	—	—	—	−0.25 (0.50)
BMI (kg/m^2^)	—	—	—	0.07 (1.02)

Values are unstandardized coefficients (B) with standard errors (SE) in parentheses.

Significance levels: * *p* < 0.05, ** *p* < 0.01, *** *p* < 0.001. Gender coded as: 1 = Male, 2 = Female. BMI, body mass index; FFM, fat-free mass; PhA, phase angle; WC, waist circumference.

Model 1 – Adjusted R^2^ = 0.096, SEE = 56.50, AIC = 5402.70, BIC = 5415.31, *F* = 53.57, *p* < 0.001.

Model 2 – Adjusted R^2^ = 0.293, SEE = 49.98, AIC = 5283.22, BIC = 5304.24, *F* = 69.19, *p* < 0.001.

Model 3 – Adjusted R^2^ = 0.310, SEE = 49.37, AIC = 5273.08, BIC = 5302.51, *F* = 45.38, *p* < 0.001.

Model 4 – Adjusted R^2^ = 0.308, SEE = 49.46, AIC = 5276.80, BIC = 5314.64, *F* = 32.34, *p* < 0.001.

The regression coefficients (β), standard errors (SE), and significance levels for each predictor are shown in the table, along with key model fit statistics for each of the four models. The predicted CAP formula was derived from Model 3 as follows:

$$\begin{array}{l} \text{Predicted}\;\mathrm{CAP}=9.637+[15.931\times \mathrm{PhA}(o)]+[1.016\times \mathrm{WC}\;(\mathrm{cm})]\\ +[1.733\times \text{Body}\;\mathrm{Fat}\;(\%)]+t[0.631\times \mathrm{Age}\;(\text{year})]\\ -[20.058\times \text{Gender}\;(\text{male}=1,\text{Female}=2)] \end{array}$$

$$\begin{array}{l} \text{Female}=[15.931\times \mathrm{PhA}\;(o)]+[1.016\times \mathrm{WC}\;(\mathrm{cm})]\\ +[1.733\times \text{Body}\;\mathrm{Fat}\;(\%)]+[0.631\times \mathrm{Age}\;(\text{year})]\\ -30.479 \end{array}$$

$$\begin{array}{l} \text{Male}=[15.931\times \mathrm{PhA}\;(o)]+[1.016\times \mathrm{WC}\;(\mathrm{cm})]\\ +[1.733\times \text{Body}\;\mathrm{Fat}\;(\%)]+[0.631\times \mathrm{Age}\;(\text{year})]\\ -10.421 \end{array}$$

To assess the model’s predictive accuracy, we calculated the Pearson correlation coefficient between the measured and predicted CAP values. The results revealed a moderate correlation (*r* = 0.563, *p* < 0.001). The mean and standard deviation for real and predicted CAP values were respectively; 277.69 ± 59.4 and 255.0 ± 33.3.

**Figure 2:**
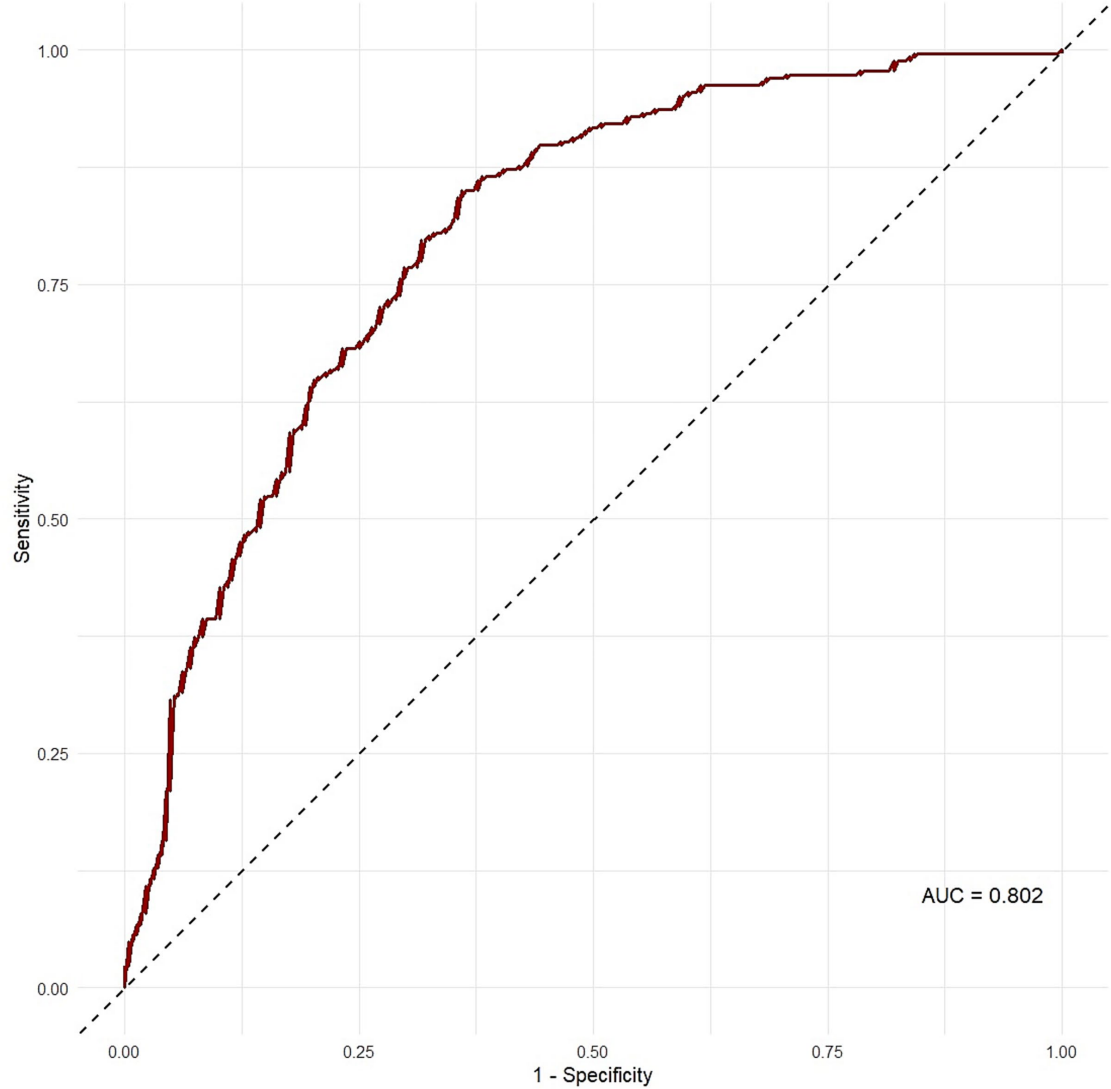
ROC curve evaluating the ability of predicted CAP values to identify SLD using the cutoff CAP ≥ 275 dB/m.

Finally, ROC analysis was performed to evaluate the discriminative performance of the predicted CAP values in identifying SLD, using the established threshold of ≥ 275 dB/m as the cut-off (Figure 2). The ROC analysis yielded an AUC of 0.802, indicating a high level of diagnostic performance. At the ≥ 275 dB/m cutoff, the model achieved a sensitivity of 47.0% and a specificity of 88.8%.

**Figure 3:**
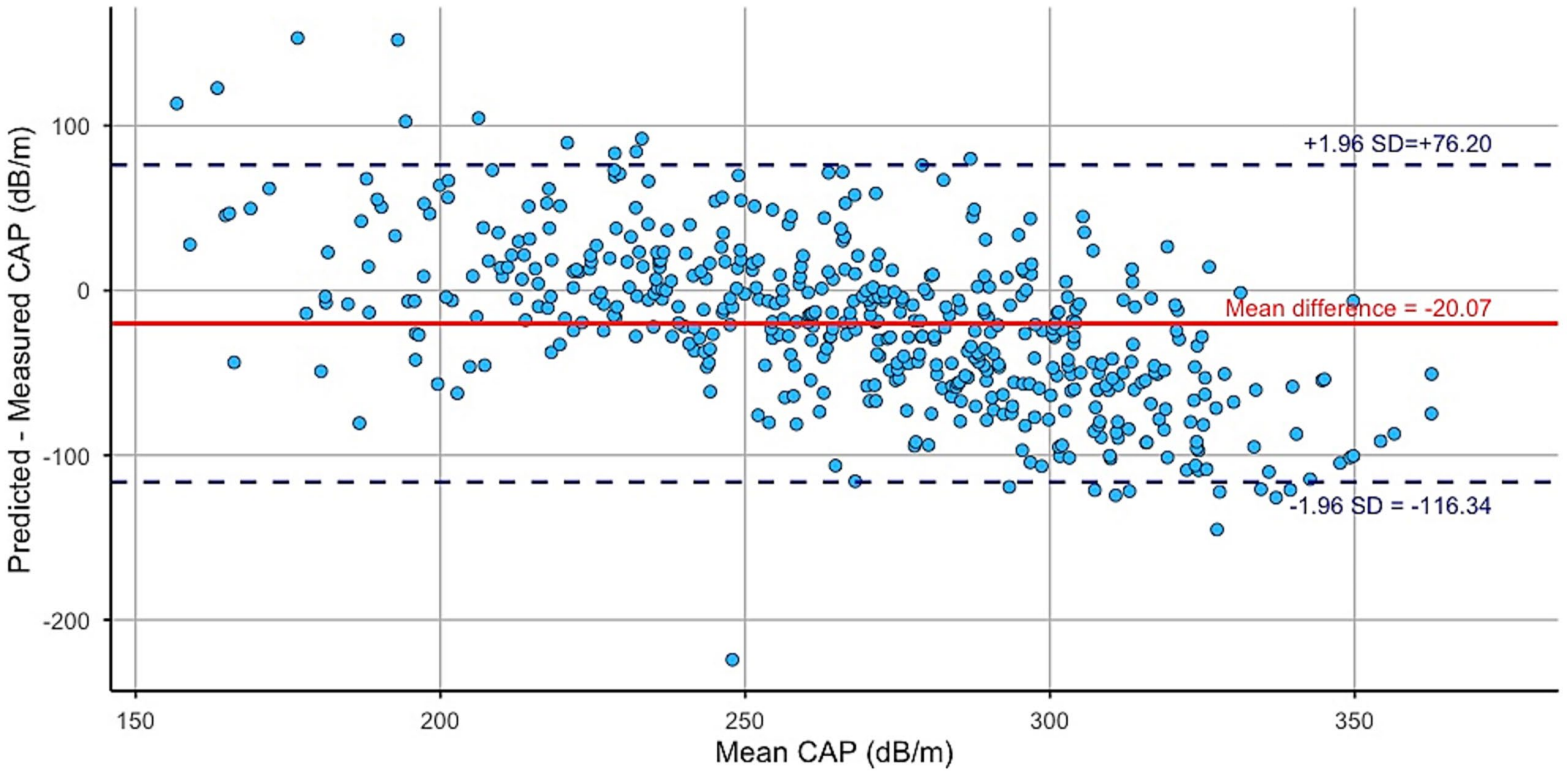
Bland–Altman plot showing the agreement between measured and predicted CAP values.

Figure 3 presents the Bland–Altman plot evaluating the agreement between the CAP values measured via VCTE and those predicted by Model 3. Model accuracy was assessed using root mean square error (RMSE) and mean absolute error (MAE), which were 53.02 dB/m and 41.59 dB/m, respectively. Agreement between measured and predicted CAP values was further evaluated using Bland–Altman analysis. The analysis demonstrated a mean difference of −20.07 dB/m with 95% limits of agreement ranging from −116.34 to 76.20 dB/m.

## 4 Discussion

This study evaluated the potential role of BIA-derived PhA in predicting SLD, using both direct measurement and predictive modeling approaches. Our findings demonstrate that individuals with SLD exhibit significantly higher PhA values across all body segments compared to those without SLD. Notably, whole-body PhA showed moderate discriminatory ability in relation to SLD status, with an optimal cut-off of 5.81°. Furthermore, PhA emerged as an independent predictor of SLD alongside waist circumference and age, while BMI and body fat percentage were not statistically significant in the logistic regression model. Rather than indicating diagnostic performance on its own, these findings suggest that PhA reflects bioelectrical properties influenced by body composition and tissue characteristics associated with hepatic steatosis, and may provide complementary information within clinical and nutritional assessments of SLD.

The observation of higher PhA values in individuals with SLD requires careful interpretation. From an impedance theory perspective, PhA represents the arctangent of the ratio between reactance (Xc) and resistance (R), reflecting cell membrane capacitance relative to conductive tissue properties (26). Traditionally, lower PhA values have been associated with cellular impairment, inflammation, and malnutrition (23). However, PhA is also influenced by body composition characteristics, including fat mass, fat-free mass distribution, and hydration status (23, 26). Chen et al. (14) demonstrated that higher PhA values were significantly associated with the risk of NAFLD in an overweight population, suggesting that individuals with higher BMI may exhibit increased PhA values due to greater body cell mass, including fat and muscle cells. In individuals with metabolic conditions characterized by increased adiposity, altered impedance properties may modify the balance between reactance and resistance, potentially resulting in relatively higher PhA values without necessarily indicating improved cellular integrity. Therefore, in this context, elevated PhA should not be interpreted as a marker of better health status but rather as reflecting bioelectrical characteristics associated with body composition alterations in SLD.

Although phase angle has traditionally been interpreted as a marker of cellular integrity and nutritional status, the finding that PhA was significantly higher in individuals with SLD challenges conventional expectations. Conventionally, lower PhA values have been associated with cellular membrane dysfunction, inflammation, and malnutrition (27–29). However, our results align with emerging evidence suggesting that higher PhA values may also reflect in-creased fat mass and preserved cell mass in metabolically active tissues (8, 15). This paradoxical relationship may be explained by the altered bioelectrical properties in obese individuals or those with higher adiposity, which can elevate PhA values due to increased reactance and relatively stable resistance. Furthermore, our regression analysis revealed that PhA remained a significant predictor of SLD even after adjusting for BMI and body fat percentage, both of which did not independently predict the presence of steatosis. This finding suggests that PhA may capture bioelectrical characteristics related to body composition that are not fully reflected by conventional anthropometric indices. Although PhA is widely recognized as a prognostic marker in chronic diseases due to its sensitivity to cell membrane integrity, emerging evidence shows that it also reflects alterations in body composition and cellular function in metabolic conditions, supporting its utility in nutritional assessment (30).

The moderate diagnostic performance of PhA (AUC = 0.646) indicates a limited-to-moderate level of discrimination when interpreted independently and supports the interpretation of PhA as a complementary indicator of metabolic and body composition features associated with SLD. The identified threshold of 5.81° may serve as a practical reference for supportive clinical assessment, although population-specific validation is required. Our regression model predicting CAP values further supports the clinical relevance of PhA, as the model incorporating PhA, waist circumference, body fat percentage, age, and gender explained 31% of the variance in CAP. Waist circumference showed the strongest correlation with CAP values, underscoring its role as a marker of visceral adiposity, a key contributor to hepatic steatosis (12). Incorporating waist circumference into our models significantly improved predictive performance, suggesting that combining PhA with simple anthropometric measures can optimize screening strategies. Age was also a significant predictor, reflecting the progressive nature of SLD with advancing age (11). In previous studies, a decline in PhA with advancing age has been consistently observed, likely due to the age-related deterioration of cell membrane integrity and body composition (13–15). In line with this, the results also demonstrated a significant negative correlation between age and PhA in both participants with and without SLD. However, when the data were stratified by age using a 40-year cut-off, individuals with SLD exhibited significantly higher PhA values compared to non-SLD individuals in both age groups. This finding indicates that the observed association between hepatic steatosis and PhA in our sample cannot be fully attributed to age-related changes alone. Accordingly, PhA may reflect metabolic alterations related to hepatic fat accumulation that persist across age groups.

In addition to age, the relationship between PhA and SLD was explored in relation to obesity status. Subgroup analyses revealed that individuals with SLD had significantly higher PhA values than those without SLD, regardless of whether they were classified as obese (BMI ≥ 30 kg/m^2^) or non-obese. These findings suggest that the association between PhA and hepatic steatosis is not solely driven by BMI-related differences in body composition. It has been demonstrated by preceding studies that BMI may not fully capture the metabolic or structural alterations relevant to liver fat accumulation (14, 15). In the present study, even among individuals who were not obese, the presence of SLD was accompanied by significantly elevated PhA values. This observation highlights the potential relevance of PhA in characterizing metabolic risk among normal-weight individuals with SLD, a subgroup that remains underrepresented in the literature.

In the multivariable logistic regression model, the non-significant contribution of BMI and body fat percentage likely stems from their conceptual and statistical overlap with waist circumference, which more directly captures visceral fat distribution. While BMI, waist circumference, and body fat percentage are related, they represent distinct indicators of adiposity—reflecting overall adiposity, central/visceral adiposity, and body composition, respectively. Previous research has established that body fat distribution, particularly visceral adiposity, plays a critical role in the development of steatotic liver disease and may influence disease progression independently of overall adiposity (31). Therefore, evaluating multiple adiposity indicators provides a more comprehensive representation of fat distribution and its metabolic implications. Despite the inherent correlation between these indices, the low variance inflation factor (VIF) values in our model confirm that each variable contributes unique variance to the prediction of SLD. This further suggests that the bioelectrical and structural information captured by PhA is distinct from mass-based measures like BMI and body fat percentage.

Due to the prevalence of SLD being significantly higher in males compared with females in our study sample, female was used as the reference category in the regression model, enabling direct interpretation of the increased risk observed in males. Moreover, predicted CAP values demonstrated a strong correlation with measured CAP, and ROC analysis demonstrated good classification performance (AUC = 0.807), underscoring the utility of combining PhA with simple anthropometric measures to estimate hepatic fat content. The Bland–Altman analysis indicated moderate agreement between measured and predicted CAP values, with a mean difference (bias) of −20.07 dB/m, indicating that the model tended to underestimate CAP values by approximately 20 dB/m on average, particularly at higher CAP levels.

Beyond its statistical performance, the clinical utility of PhA lies in its potential integration into routine nutritional screening—particularly by dietitians who often serve as the first point of contact for individuals with metabolic risk. Using a cut-off value of 5.81°, whole-body PhA identified individuals with SLD with a sensitivity of 69.6% and a specificity of 53.3%, indicating a reasonable balance between true positives and false positives. This means that a substantial proportion of individuals with early steatotic changes can be detected using a quick, non-invasive BIA device. Indeed, in many clinical scenarios, false negatives may carry more weight than false positives, depending on the priority of not missing a diagnosis (32). For dietitians, this offers an evidence-based opportunity to flag individuals at risk—even in the absence of biochemical parameters or imaging-based confirmation—and refer them for further evaluation by a primary care physician, and if necessary, by a hepatologist afterwards. Such early identification may support timely diagnosis, lifestyle modification, and prevention of disease progression, ultimately contributing to better long-term outcomes and reduced healthcare burden.

Given the non-invasive nature of PhA measurement (9), its association with SLD presence, and its predictive strength in regression models, PhA may serve as a valuable adjunct in routine nutritional and metabolic assessments. It could be especially useful in primary care settings or regions lacking access to advanced imaging modalities such as FibroScan®. Importantly, PhA is not intended to replace imaging-based assessment of hepatic steatosis but to complement existing clinical information within an integrated evaluation framework.

This study has several strengths, including a relatively large sample size, use of standardized PhA measurement techniques, and integration of multiple body composition parameters. Furthermore, the statistical modeling approach provides a practical tool for estimating hepatic fat content. However, certain limitations must be acknowledged. The retrospective design restricts causal inferences, and liver biopsy, the gold standard for diagnosing steatosis, was not available. PhA measurements may also be influenced by hydration status or unmeasured comorbidities. Furthermore, glycemic markers were not consistently available in the retrospective records and therefore could not be included in the multivariable analysis. Additionally, due to incomplete and non-standardized alcohol consumption data inherent to the retrospective design, reliable differentiation between disease-etiology–based subgroups were not feasible. External validation of the proposed PhA cut-off is needed to generalize findings to broader populations.

Future prospective studies are warranted to validate the clinical applicability of PhA across diverse populations and to refine cut-off values according to age, sex, and body composition profiles. Longitudinal research could also explore whether changes in PhA over time correspond to improvements in liver health or treatment response. Ultimately, integrating PhA with other non-invasive clinical and anthropometric markers may enhance the contextual assessment of hepatic steatosis risk.

## 5 Conclusion

PhA, measured via bioelectrical impedance analysis, represents a practical and non-invasive parameter reflecting bioelectrical properties related to body composition that are associated with SLD, particularly in settings where access to advanced imaging technologies is limited. Our findings indicate that PhA is associated with hepatic steatosis beyond the effects of age or obesity; however, its clinical relevance should be interpreted within a broader clinical and nutritional assessment framework rather than as a standalone diagnostic marker. Incorporating PhA into routine nutritional assessments conducted by dietitians may help contextualize metabolic risk and support individualized nutritional counseling, even in the absence of biochemical or radiological data. When integrated with other clinical and anthropometric information, such an approach may contribute to timely multidisciplinary evaluation, facilitate lifestyle-based interventions, and support strategies aimed at reducing disease progression and long-term healthcare burden.

## Data Availability

The original contributions presented in the study are included in the article/supplementary material, further inquiries can be directed to the corresponding author.
